# How to design a dose-finding study using the continual reassessment method

**DOI:** 10.1186/s12874-018-0638-z

**Published:** 2019-01-18

**Authors:** Graham M. Wheeler, Adrian P. Mander, Alun Bedding, Kristian Brock, Victoria Cornelius, Andrew P. Grieve, Thomas Jaki, Sharon B. Love, Lang’o Odondi, Christopher J. Weir, Christina Yap, Simon J. Bond

**Affiliations:** 1 Cancer Research UK and UCL Cancer Trials Centre, University College London, 90 Tottenham Court Road, London, W1T 4TJ UK; 2 MRC Biostatistics Unit Hub for Trials Methodology Research, University of Cambridge, Cambridge Institute of Public Health, Forvie Site, Robinson Way, Cambridge Biomedical Campus, Cambridge, CB2 0SR UK; 3 Roche Pharmaceuticals, Hexagon Place, Falcon Way, Shire Park, Welwyn Garden City, AL7 1TW UK; 4 Cancer Research UK Clinical Trials Unit, Institute of Cancer and Genomic Sciences, University of Birmingham, Edgbaston, Birmingham, B15 2TT UK; 5 School of Public Health, Imperial College London, 68 Wood Lane, London, W12 7RH UK; 6 UCB Pharmaceuticals Ltd, 208 Bath Road, Slough, SL1 3WE UK; 7 Department of Mathematics and Statistics, Fylde College, Lancaster University, Fylde Avenue, Bailrigg, Lancaster, LA1 4YF UK; 8 Oxford Clinical Trials Research Unit, Centre for Statistics in Medicine, NDORMS, University of Oxford, Botnar Research Centre, Windmill Road, Oxford, OX3 7LD UK; 9 MRC Clinical Trials Unit, University College London, 90 High Holborn, London, WC1V 6LJ UK; 10 Edinburgh Clinical Trials Unit, Usher Institute of Population Health Sciences, University of Edinburgh, Nine Edinburgh Bioquarter, 9 Little France Road, Edinburgh, EH16 4UX UK; 11 National Institute for Health Research Cambridge Clinical Trials Unit, Cambridge University Hospitals NHS Foundation Trust, Addenbrooke’s Hospital, Hills Road, Cambridge Biomedical Campus, Box 401, Coton House Level 6, Cambridge, CB2 0QQ UK

**Keywords:** Adaptive designs, Continual reassessment method, Dose escalation, Dose-finding, Maximum tolerated dose, Phase I trials

## Abstract

**Introduction:**

The continual reassessment method (CRM) is a model-based design for phase I trials, which aims to find the maximum tolerated dose (MTD) of a new therapy. The CRM has been shown to be more accurate in targeting the MTD than traditional rule-based approaches such as the 3 + 3 design, which is used in most phase I trials. Furthermore, the CRM has been shown to assign more trial participants at or close to the MTD than the 3 + 3 design. However, the CRM’s uptake in clinical research has been incredibly slow, putting trial participants, drug development and patients at risk. Barriers to increasing the use of the CRM have been identified, most notably a lack of knowledge amongst clinicians and statisticians on how to apply new designs in practice. No recent tutorial, guidelines, or recommendations for clinicians on conducting dose-finding studies using the CRM are available. Furthermore, practical resources to support clinicians considering the CRM for their trials are scarce.

**Methods:**

To help overcome these barriers, we present a structured framework for designing a dose-finding study using the CRM. We give recommendations for key design parameters and advise on conducting pre-trial simulation work to tailor the design to a specific trial. We provide practical tools to support clinicians and statisticians, including software recommendations, and template text and tables that can be edited and inserted into a trial protocol. We also give guidance on how to conduct and report dose-finding studies using the CRM.

**Results:**

An initial set of design recommendations are provided to kick-start the design process. To complement these and the additional resources, we describe two published dose-finding trials that used the CRM. We discuss their designs, how they were conducted and analysed, and compare them to what would have happened under a 3 + 3 design.

**Conclusions:**

The framework and resources we provide are aimed at clinicians and statisticians new to the CRM design. Provision of key resources in this contemporary guidance paper will hopefully improve the uptake of the CRM in phase I dose-finding trials.

**Electronic supplementary material:**

The online version of this article (10.1186/s12874-018-0638-z) contains supplementary material, which is available to authorized users.

## Background

Phase I trials are conducted to find the maximum tolerated dose (MTD) of a new drug or treatment. The MTD is defined as “…the dose expected to produce some degree of medically unacceptable dose-limiting toxicity…in a specified proportion…of patients” [1]. The “specified proportion” in this definition is commonly known as the target toxicity level (TTL).

Most phase I trials use rule-based approaches, such as the 3 + 3 design [2, 3], to identify the MTD [4, 5]. Under the 3 + 3 design, cohorts of three patients are assigned to increasing dose levels until one or more dose-limiting toxicities (DLTs) is observed. If one out of three patients has a DLT, a further three patients are assigned to the current dose. If two or more patients out of three or six patients at the current dose experience a DLT, the trial is terminated and the dose below this level is declared the MTD. The 3 + 3 design uses only data at the current dose to choose the next dose and MTD, resulting in uncertainty around the estimated DLT risks at each dose. Furthermore, as no TTL is specified by investigators when using the 3 + 3 design, the identified MTD often has a true risk of causing severe toxicity far different to what clinicians may deem acceptable for the treatment under investigation. These and other drawbacks in rule-based designs have been identified and reported [6, 7]. The Medical Research Council (MRC) Network of Hubs for Trials Methodology Research’s Adaptive Designs Working Group published a short note on why the 3 + 3 design, and A + B designs in general, should not be used for dose-finding studies. They provide guidance on better designs and software for conducting dose-finding studies [8].

Model-based designs are an alternative to rule-based designs [9]. They use a statistical model to estimate the relationship between dose and DLT risk, which then informs dose escalation decisions. The model is also used to identify the MTD, which is defined relative to a TTL explicitly specified by investigators before the trial. The most well-known model-based design is the continual reassessment method (CRM) [10]. The CRM combines all available trial data, with available information from clinicians and past trials, to estimate the MTD. Many studies have compared the CRM to the 3 + 3 design and found that the CRM is more likely to recommend the correct MTD and dose more trial patients close to the MTD [11–15].

Although first proposed nearly 30 years ago, the uptake of the CRM in mainstream clinical research has been unfortunately slow [3–5, 16]. Garrett-Mayer [17] published a tutorial paper on the CRM, which described the design and used two simulated trials to illustrate how studies may be conducted. Since then, the landscape has changed: a handful of trials have used the CRM in practice [5]; new software has been developed; further recommendations have been provided, based on both theoretical research and practical experience [18, 19]; and regulatory agencies have updated guidance documents to explicitly mention adaptive designs for clinical trials [20, 21]. Several barriers to the implementation of the CRM have been formally identified too. These include a lack of expertise, both in the clinical and statistical communities, a lack of user-friendly software, and a fear that recommendations from a model-based design cannot be overridden by clinicians [22–24]. To help overcome these barriers and provide up-to-date resources for investigators, we detail how to design and conduct a phase I dose-finding study using the CRM. We describe the key components of the CRM, illustrate a framework to structure the design process, and list the decisions the trial team should make. We provide recommendations for fine-tuning the design and describe available software to assist clinicians and statisticians in doing this. We also provide text and tables that can be customised and inserted into a trial protocol. We conclude by illustrating two real dose-finding trials that used the CRM, describing how they were designed and conducted, and compare their performance to the traditional 3 + 3 design.

## Methods

Here we describe and discuss the key parameters that are needed to set up and run a CRM trial. These are: Number of doses; Target Toxicity Level; Dose-toxicity model; Dose-toxicity skeleton; Method of inference; Decision rules; Sample size and cohort size; Safety modifications; and Stopping rules.

### Number of doses

**Fig. 1:**
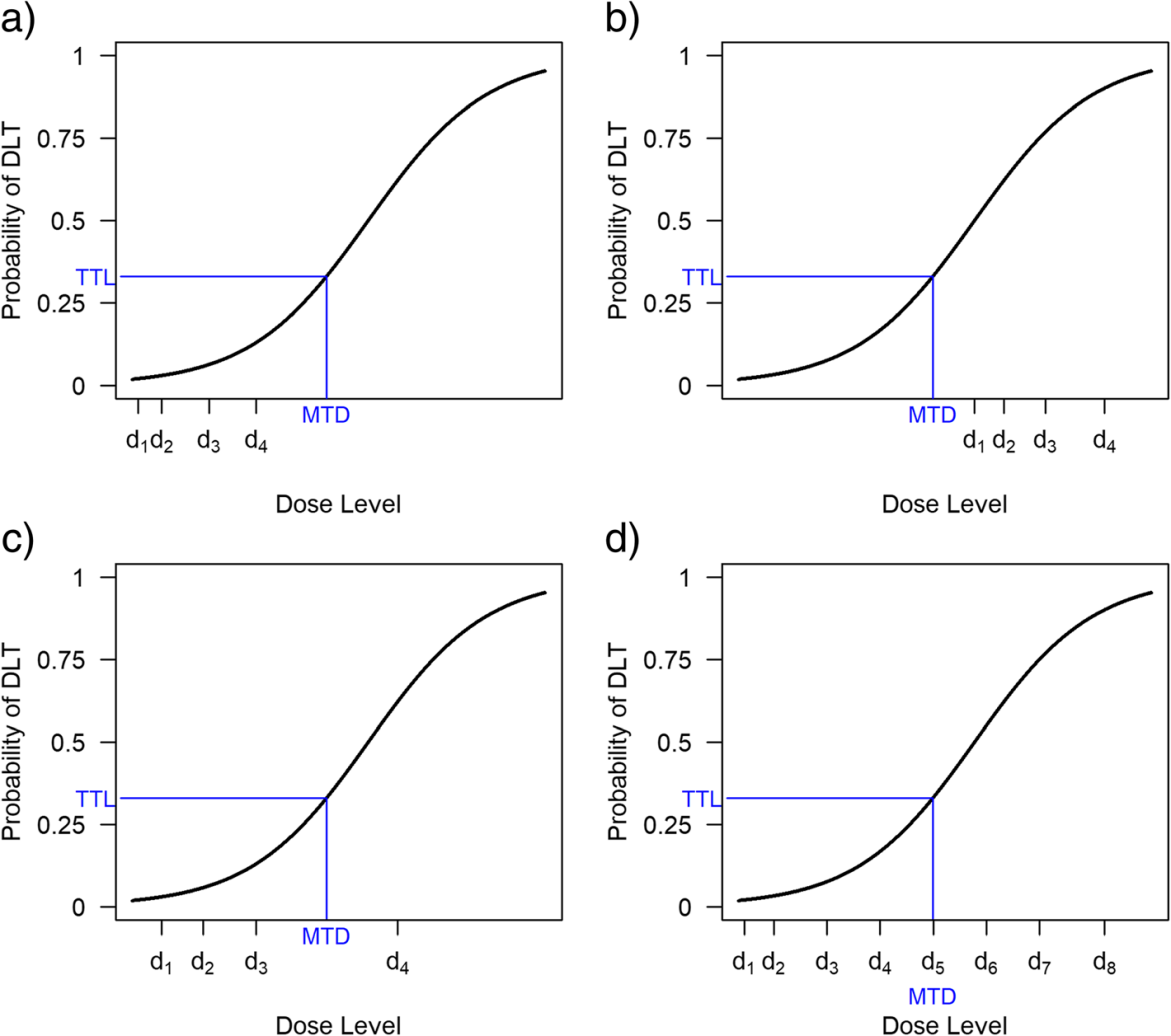
Number and spacing of doses for a dose-finding trial. The doses in Fig. 2(a) are too low to estimate the MTD, whereas those in Fig. 2(b) are too high. In Fig. 2(c), the target dose lies between two dose levels, so patients will be assigned alternately to an overdose level and an underdose level; the final MTD will likely be at one of these levels. Figure 2(d) illustrates a situation with several dose levels available in the region of the MTD.

Statistical and practical considerations underlie the choice of how many and which doses to study. The most important statistical consideration is whether the doses and dose range under investigation are likely to allow an accurate MTD estimate. Figure 1 shows how different dose range choices affect MTD selection under the same dose-toxicity relationship. Too few doses may mean the MTD will be poorly estimated, whereas too many doses can hinder dose escalation towards the MTD.

Which doses are investigated in a trial is often determined by practical restrictions. For oral treatments, for example, dose levels may increase based on number of tablets. If the treatment is produced specifically for the study (as in first-in-man studies), finances may limit how many dose levels can be manufactured. However, techniques such as allometric scaling can be used to choose which doses should be studied [25]. In a review of 197 phase I trials published between 1997 and 2008, the median number of dose levels explored was five (range 2–12) [26].

### Target toxicity level

The acceptable chance of a patient experiencing a DLT (the TTL) must be set before the trial starts. The TTL depends on the disease, treatment under investigation, availability of alternative treatment options, patients’ performance status, and likely associated adverse events included in the definition of DLT. The TTL is determined by clinical expertise, evidence from previous studies, and guidance from the trial statistician. Often the TTL is set between 20 and 35%, but some studies have set the TTL as high as 40% [27, 28].

### Dose-toxicity model

**Fig. 2:**
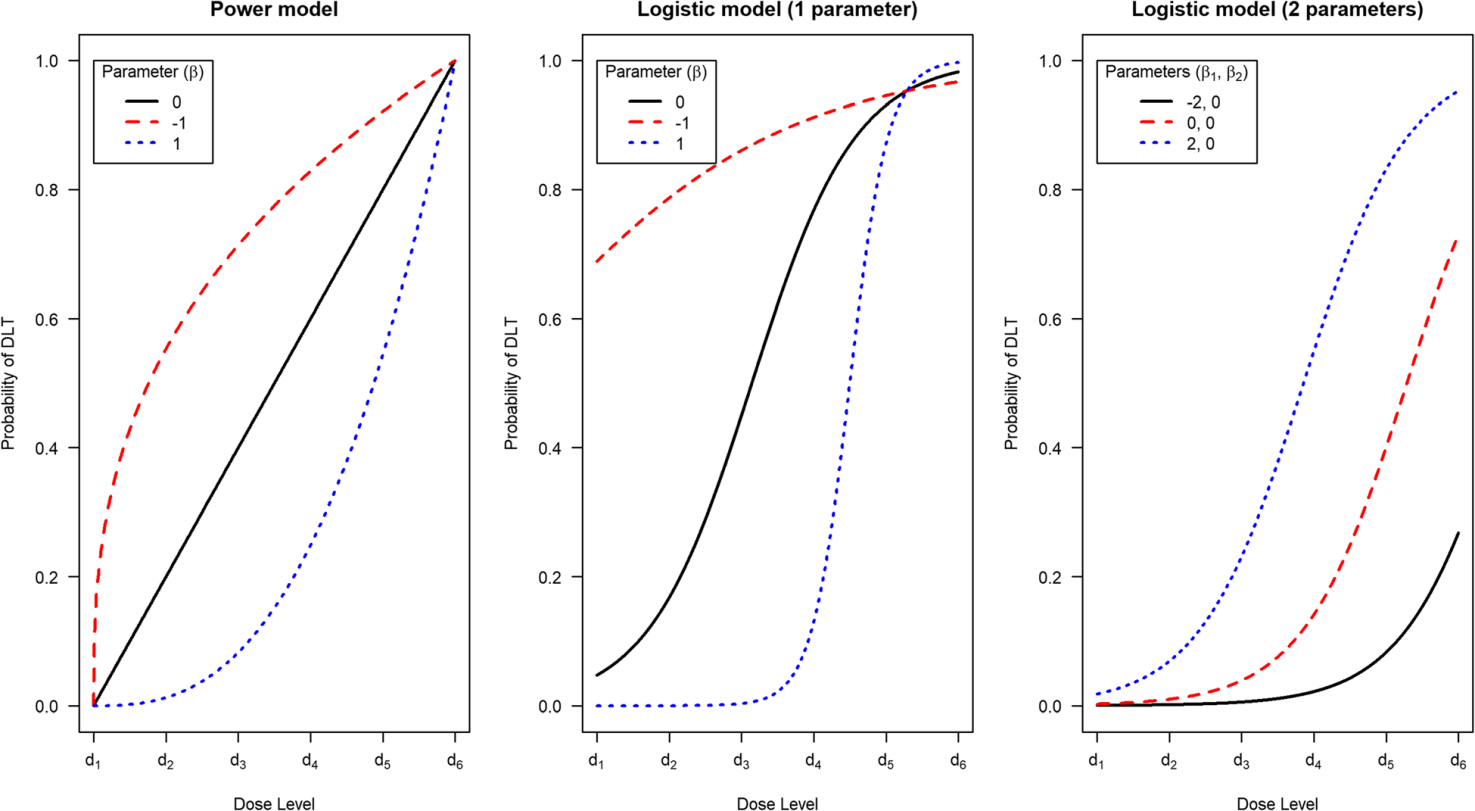
Dose-toxicity relationships for different dose-toxicity functions with varying parameter values

We need to state how we will model the relationship between dose and the risk of observing a DLT. The dose-toxicity model describes the probability of a patient experiencing a DLT at a given dose (the dose-toxicity relationship). The model is a fixed mathematical function that is monotonically increasing in dose, i.e. as the dose increases, so does the probability of observing a DLT. The model is written as *F*(*β*, *d*), where *F*(·,·) is the chosen dose-toxicity function (see Table 1), *β* is a vector of one or more parameters that alters the shape of the dose-toxicity relationship, and *d* is the *dose label* for a particular drug dose. Figure 2 shows some dose-toxicity relationships for different function choices and parameter values.

**Table 1:** Common choices for dose-toxicity models and resultant dose labels for the CRM

Model name	Model (*F*(*β*, *d*))	General form of dose labels (*d*_*i*_)	Choice of β* (prior mean or median)	Dose labels given β* (*d*_*i*_)
Power (empiric)	*d* ^*exp*( *β*)^	${p}_i^{\frac{1}{\mathit{\exp}\left(\beta \right)}}$	*β* = 0	*p*_*i*_
One-parameter logistic	$\frac{\mathit{\exp}\left(3+\mathit{\exp}\ \left(\beta \right)\ d\right)}{1+\mathit{\exp}\left(3+\mathit{\exp}\ \left(\beta \right)\ d\right)}$	$\frac{\mathit{\ln}\left(\frac{p_i}{1-{p}_i}\right)-3}{\mathit{\exp}\left(\beta \right)}$	*β* = 0	$\mathit{\ln}\left(\frac{p_i}{1-{p}_i}\right)-3$
Two-parameter logistic	$\frac{\mathit{\exp}\left({\beta}_1+\mathit{\exp}\ \left({\beta}_2\right)\ d\right)}{1+\mathit{\exp}\left({\beta}_1+\mathit{\exp}\ \left({\beta}_2\right)\ d\right)}$	$\frac{\mathit{\ln}\left(\frac{p_i}{1-{p}_i}\right)-{\beta}_1}{\mathit{\exp}\left({\beta}_2\right)}$	*β*_1_ = 0, *β*_2_ = 0	$\mathit{\ln}\left(\frac{p_i}{1-{p}_i}\right)$

Notation: *p*_*i*_ = skeleton probability of DLT at *i*^th^ dose level; *d*_*i*_ = dose label for for *i*^th^ dose level

### Dose-toxicity skeleton

**Fig. 3:**
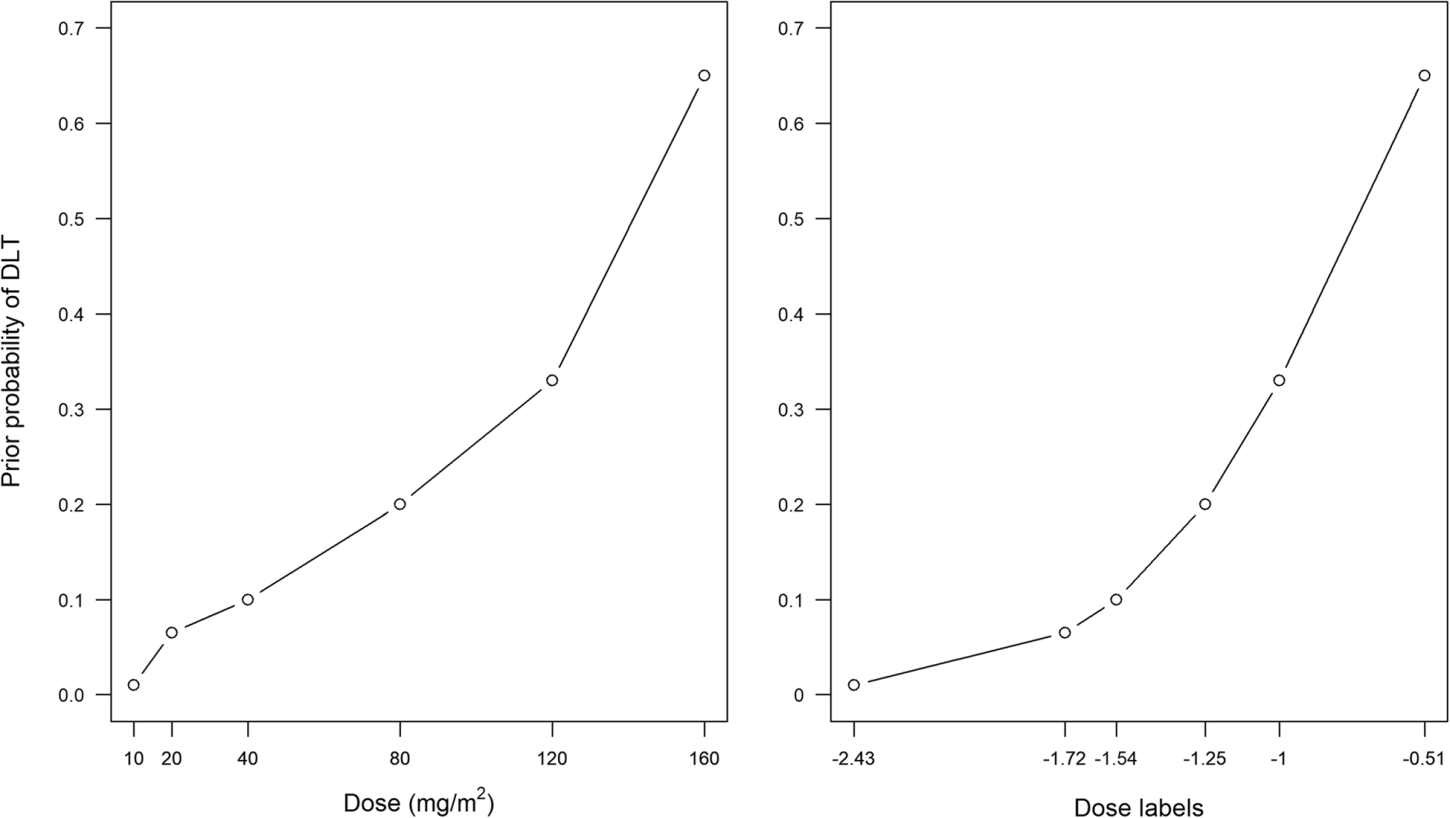
Example of transforming drug-specific doses to dose labels using prior skeleton probabilities of DLT risk. Two-parameter logistic model with prior average parameter values β_1_ = 2 and β_2_ = 1 (see Table A1 in Additional file 1: Appendix A for calculations).

Selecting a model for the dose-toxicity relationship can seem daunting at first. However, we can ensure our chosen model has a sensible shape over the dose levels of interest by specifying a *skeleton*. The skeleton is the set of expected DLT probabilities at the dose levels of interest and is specified by one or more clinicians before the trial. For a trial with *k* dose levels, the clinical team specifies a prior average estimate for the probability of DLT at each dose. These are denoted here as *p*_1_, …, *p*_*k*_ (the skeleton), and are only constrained to be monotonically increasing and distinct from one another. For dose-toxicity model *F*(·,·), the *dose label* for the *i*^*th*^ dose is then *d*_*i*_, such that *p*_*i*_ = *F*(β*, *d*_*i*_). Here, β* can be the prior mean or median of the model parameter *β*. Using dose labels ensures the model fits the skeleton well before the trial; the actual dose scale of the drug does not matter. Common model choices, prior reference values, and resultant dose labels are given in Table 1. An example transformation from drug-specific doses to dose labels is shown in Fig. 3 (calculations given in Table A1 (Additional file 1: Appendix A)).

Ultimately, the choice of model and skeleton are not unique, as different pairings of dose-toxicity model and skeleton can lead to identical dose-escalation recommendations after a given sequence of observations [18]. With regards to the one-parameter logistic model, the value of the fixed intercept (set to 3 in Table 1) does not affect the shape of the dose-toxicity model. However, the value of the fixed intercept affects the resultant dose labels and the credible intervals. In designing a trial of capecitabine in combination with epirubicin and cyclophosphamide in patients with advanced breast cancer, Morita [29] showed that changing the value of the intercept shifted the greatest uncertainty in DLT risk from the lowest dose to the highest dose. Therefore, if using the one-parameter logistic model, the intercept can be chosen to give prior uncertainties around dose levels that matches clinical expectations.

Several papers have investigated how the number of model parameters affects a CRM design’s theoretical properties and operating characteristics, including the chance of estimating each dose as the MTD, percentage of patients allocated to each dose level, average sample size, and average proportion of patients who will experience a DLT [30–33]. Using a one- or two-parameter model affects how strongly data at lower doses influence the next dose choice. A one-parameter model is more likely to make recommendations that lead to faster escalation through the doses, resulting in a more efficient trial, but put participants at higher risk of experiencing DLTs. A two-parameter model is likely to better estimate the shape of the entire dose-toxicity relationship [34], but less efficiently identify the MTD; it may take longer to reach the MTD since two parameters must be estimated, and there may be difficulties fitting the model or obtaining consistent estimates of model parameters [31].

Although we cannot know the true shape of the dose-toxicity relationship, the dose recommendations made after each cohort will get closer to the MTD. Certainly with a one-parameter model, we will reach a reliable estimate of the MTD (and its probability of DLT), even if our estimates for doses further away are inaccurate. This result is insensitive to the model and dose labels used [35], although the skeleton probabilities should be spaced reasonably well apart. A skeleton with prior DLT probabilities too close together will lead to slower dose escalation, and a skeleton with prior DLT probabilities too far apart will lead to poor convergence towards the MTD [18]. Lee and Cheung [36] and Cheung [18] proposed choosing a skeleton by specifying the TTL and an *indifference interval*. This is a probability interval within which the clinician is happy for the DLT probability of the MTD to fall. For example, a TTL of 25%, give or take 5%, gives an indifference interval of [20, 30%]. An example of choosing a skeleton using the indifference interval approach is given in Additional file 1: Appendix B.

Once the number of dose levels, the TTL, dose-toxicity model and skeleton have been specified, other components of the trial design can be discussed.

### Inference

To make decisions by combining accruing trial data and other evidence, we must state how we intend to make statistical inferences on the model parameter(s), and therefore the estimated DLT probability at each dose.

A likelihood-based approach can be used; the model parameter(s) (denoted *β* previously) are estimated by applying maximum likelihood methods to the trial data. All major statistical software packages can perform these analyses. Maximum likelihood methods can only be used with heterogeneous response data (i.e., at least one DLT and one non-DLT response) to calculate parameter estimates [35]. To obtain heterogeneous response data, the design is split into two stages. Individual patients, or small cohorts of patients, are sequentially assigned to increasing dose levels until the first DLT is observed. The likelihood model-based design then takes over; a maximum likelihood estimate of the model parameter is used to update the estimated DLT probabilities [37].

Another approach is to use Bayesian inference. A prior probability distribution is assigned to the model parameter(s), which translates to assigning a prior belief (and some uncertainty) to the probability of DLT at each dose. Prior beliefs and uncertainties can be derived from different information sources, such as pre-clinical work, clinical opinion [29, 38] and data from previous trials [39]. Where relevant prior data are unavailable, appropriate vague priors can be used [40–42]. If each dose is considered equally likely to be the MTD before the trial, a “least informative” prior can be obtained to reflect this belief [40].

Data from patients in the trial are used to update the prior distribution on the model parameter(s), which then gives a posterior distribution for the model parameter(s) and therefore posterior beliefs for the probability of DLT at each dose. These posterior probabilities are used to make dose escalation decisions. By assessing a design’s operating characteristics with a specific prior in a variety of scenarios, the prior distribution can be recalibrated until the model makes recommendations for dose escalations and the MTD that the trial team are happy with [43, 44]. This iterative process helps ensure the design is appropriately configured for the trial.

### Decision rules

Under a CRM approach, we do not assign the next patient(s) to a dose level based only on the proportion of patients with DLTs at the current dose level. Using a model allows borrowing of information across dose levels. We learn more about the toxicity risk of other dose levels based on accrued data, which improves trial efficiency. We may adapt the dose for the next patient or cohort by estimating the probability of DLT for each dose level, whether from a likelihood-based or Bayesian approach, and then choosing the dose level using a specified decision rule. Possible decision rules include choosing the dose with an estimated probability of DLT closest to the TTL or, more conservatively, choosing the dose with an estimated probability of DLT closest to, but not greater than, the TTL. The first option allows quicker escalation towards the true MTD, but may expose more patients to overdoses. The second option reduces the chance of overdosing patients, but may take longer to escalate towards the true MTD.

### Sample size and cohort size

Planned sample sizes in phase I trials are generally dictated by practical constraints, such as the number of centres, projected recruitment rates, and number of dose levels, rather than statistical constraints related to type I error rate or minimum power for testing a specific hypothesis. Cheung [45] proposed formulae that use a target average percentage of correctly selecting the MTD (say, 50% of the time) to obtain a lower bound for the trial sample size. We can then use simulations to assess the design’s operating characteristics with the sample size fixed at this lower bound, and revise the sample size if necessary. We suggest specifying a lower bound based on Cheung’s work and a practical upper bound in grant applications and trial protocols.

Once a reasonable sample size has been specified, investigators can decide how many patients should be dosed at each recommended dose before a dose-escalation decision is made; this is called the *cohort size*. A cohort size of one patient will provide better operating characteristics than dosing several patients simultaneously at a dose level, although the latter can reduce the trial duration [46] and still perform better than the 3 + 3 design [47]. Regulatory requirements may also affect cohort sizes. For example, we may be required to observe safety data from the first patient before dosing other patients in that cohort. Following the recent phase I trial disasters of TeGenero’s monoclonal antibody TGN1412 and Bial’s fatty acid amide hydrolase inhibitor BIA 10–2474, measures for monitoring patients must be in place if cohorts of two or more patients are used [48, 49].

### Safety modifications

Modifications to trial designs and dose-escalation rules can easily be made to prevent overdosing patients and ensure a trial design has sensible operating characteristics. For example, the original CRM approach proposed dosing the first patient at the prior MTD guess, but many trialists propose dosing the first patient at a level lower than this (possibly even the lowest [47]). For the Viola trial [50], which used the CRM to find the MTD of lenalidomide and azacitidine in patients with relapsed acute myeloid leukemia post allogenic stem cell transplant, the middle (fourth) of seven possible doses was considered to be the prior MTD. However, the study team chose to start at the dose below this level (third) [51]. Some have suggested not skipping untested dose levels when escalating to reduce the number of patients exposed to toxic doses [47, 52–54]. Faries [52] also enforced coherent dose-escalation: if the last patient had a DLT, the next patient would not receive a dose higher than that of the last patient, even if the model recommended it. Under most trial setups of the CRM, coherence is guaranteed [55], though this should be checked in simulations.

### Stopping rules

We need to state criteria for stopping the trial before the maximum number of patients have been treated. Early termination can be considered if the MTD is judged to be outside the planned set of doses (i.e., all doses are too toxic or all doses have a probability of a DLT well below the TTL), or if adding more patients into the trial is unlikely to yield information that would change the current MTD estimate [56]. Investigators may stop a trial if either: a fixed number of patients have been consecutively dosed at one dose level [49]; the estimated probability of all dose levels having a DLT rate above (or below) the TTL is at least 90% [57, 58]; the width of the likelihood-based confidence interval or Bayesian credible interval for the MTD reaches a particular level [10]; the probability that the next *m* patients to be dosed in the trial will be given the same dose level, regardless of DLT outcomes observed, exceeds some level (e.g., 90%) [10, 56, 59]; or any combination of these [54]. If stopping a trial after a fixed number of patients, the number should be chosen based on some probabilistic criterion, e.g. if 10 consecutive patients receive the same dose level, then we are at least 90% certain that the current dose is the MTD. Therefore, using probabilistic approaches for early termination, or justifying other stopping rules using probabilities, is encouraged. In the Viola trial, the trial would be stopped early for toxicity if the chance that the risk of DLT at the lowest dose was at least 10% above the TTL exceeded 72%; this was tailored based on the clinicians’ wishes to stop the trial if they saw an unexpected number of DLTs at the lowest dose [51].

### Evaluating designs by simulation

Once an initial setup for the design has been specified according to the parameters above, we need to understand a design’s *operating characteristics* under different dose-toxicity scenarios. This is best achieved by the trial statistician simulating many trials under each scenario. The objectives of these simulation studies are to:

- demonstrate that a design has satisfactory operating characteristics by the trial team’s standards, or give results that the trial team can use to discuss and modify the design;
- form a comprehensive comparison of alternative designs, including the 3 + 3 design and a benchmark design [60];
- clearly identify the best parameter choices;
- justify the sample size; and
- give information for use in grant applications and the protocol.

The operating characteristics assessed should include the probability of selecting each dose as the MTD, number/proportion of patients given each dose, number of DLTs per dose and in total, expected sample size, and expected study duration.

**Fig. 4:**
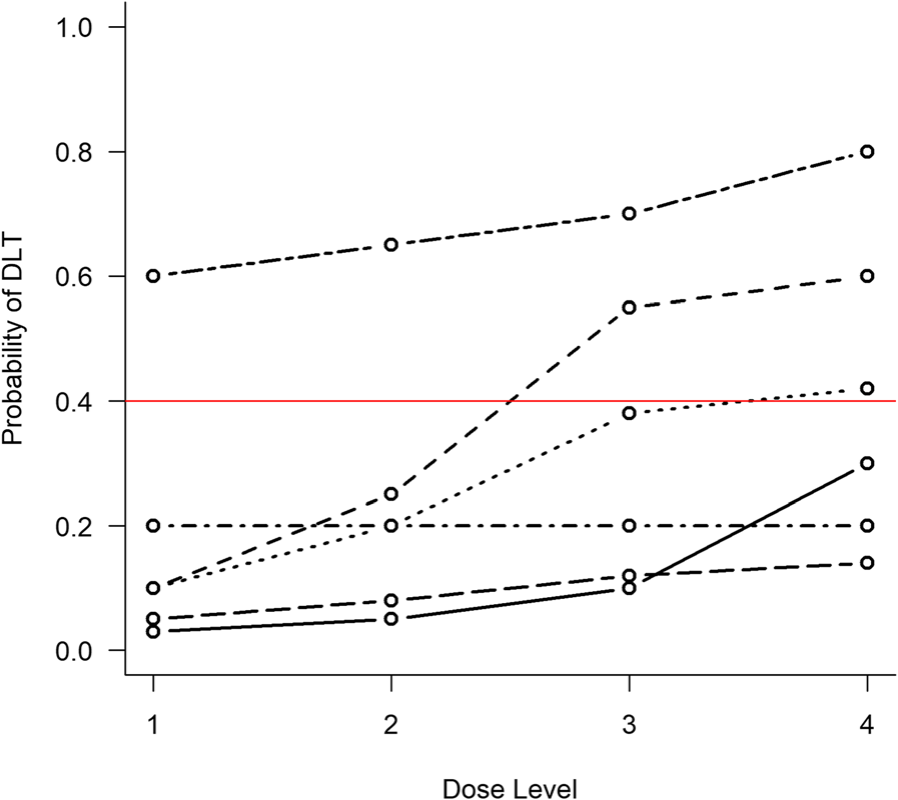
Dose-toxicity scenarios explored in the Matchpoint trial. Red line indicates TTL of 40%

The dose-toxicity scenarios used in the simulation study should include: scenarios where each dose is in fact the MTD; two extreme scenarios, in which the lowest dose is above the MTD and the highest dose is below the MTD; and any others that clinicians believe are plausible. It is worthwhile considering unlikely but extreme scenarios (e.g., first few doses are far below the MTD, then next highest far above the MTD) to see how the trial design behaves. For designing the CHARIOT trial, Frangou et al. [61] considered true dose-toxicity curves over six dose levels (schedules), which included scenarios where the TTL of 25% was found at an exact dose, or was located between two dose schedules. Brock et al. [27], when conducting pre-trial simulations for the Matchpoint trial, looked at six dose-toxicity scenarios over four dose levels; these included two scenarios where the MTD (the dose with an expected risk of DLT equal to 40%) was located between two dose levels (Fig. 4).

The pre-trial simulation studies should be conducted following recommended best practices [62, 63]:

- Create a detailed simulation plan, including expected setup time, resources required, and overall time needed to obtain results [64, 65];
- Record the random seed used, to allow replication;
- Generate a wide range of scenarios to investigate;
- Specify the number of simulation replications needed to reduce variability in the operating characteristics. Although there is no ideal number, the larger the number of simulations, the lower the variability in results;
- Run all competing designs (including a 3 + 3 design) across all simulation scenarios to compare the operating characteristics of interest.

**Fig. 5:**
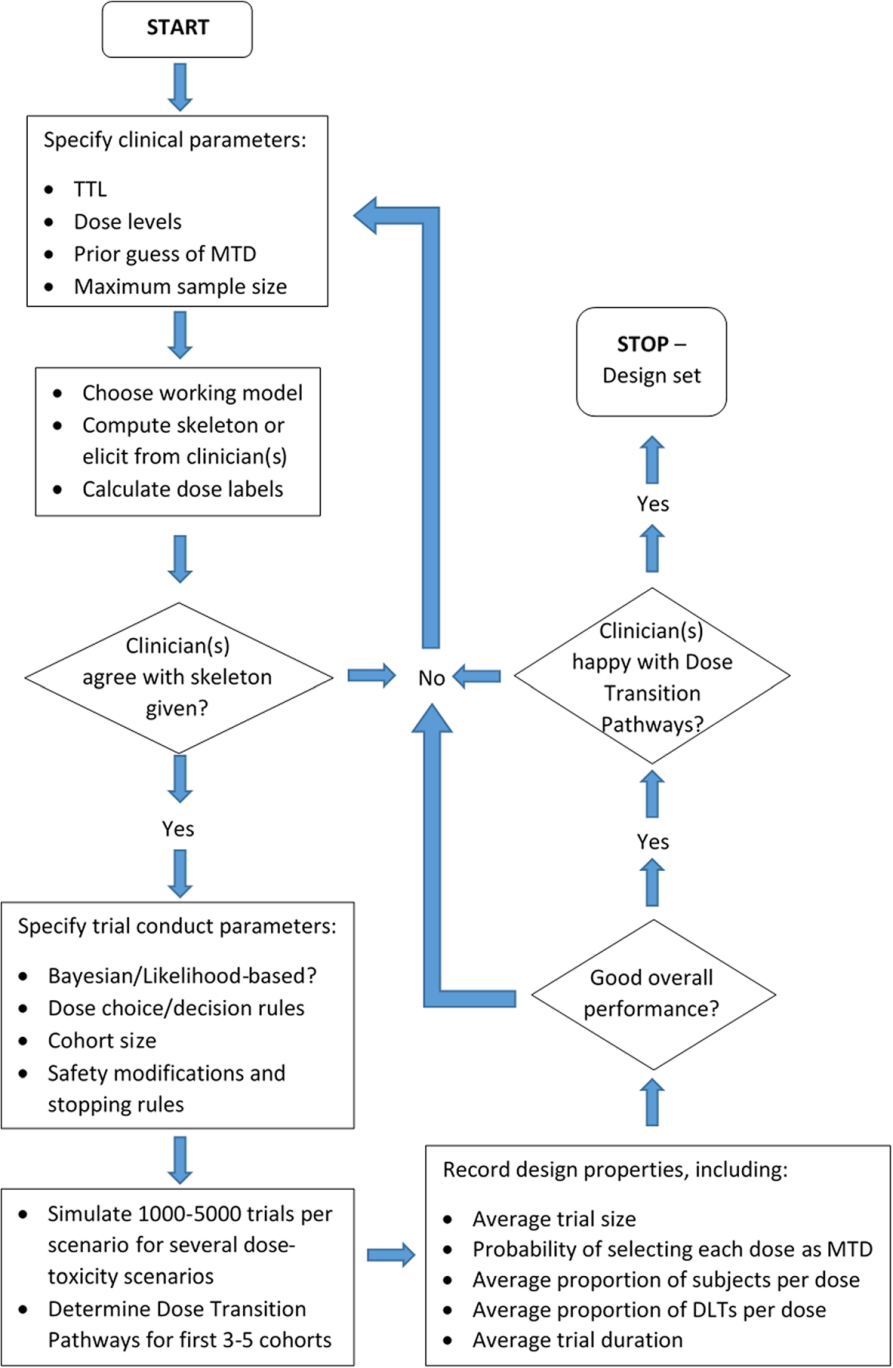
Flowchart of the trial design process using the CRM

In addition to simulations, we can assess the model recommendations based on a possible set of trial data. We can calculate in advance every feasible sequence of doses resulting from different DLT/non-DLT responses from patients in the next few cohorts; these are known as *dose transition pathways* [51]. The trial team can generate dose transition pathways to see if the design exhibits undesirable behaviour, such as not stopping the trial despite observing excessive toxicity at low doses. The design may then be recalibrated to provide dose transition pathways that clinicians and the trial team are happy with. Yap et al. [51] describe how they used dose-transition pathways to design the Viola trial. Figure 5 illustrates the trial design process in its entirety. The iterative structure shows the discussions that are required to decide on different aspects of the design, and how and when they should be evaluated.

### Finalising the design

Once the trial design has been agreed, the pre-trial simulations should be documented, detailing the set-up specifications, which designs were compared under which scenarios, and an easily interpretable summary of the design’s main features. This report can be included in the protocol appendix or statistical analysis plan, or can be a separate report that is formally acknowledged in the protocol and statistical analysis plan and stored in the trial master file. We provide a general description of the CRM that can be used in trial protocols in Additional file 1: Appendix C. The target audiences for the simulation report are internal project teams and the research ethics committee. For some dose-finding trials, simulation reports may need to be submitted to regulators.

### Trial conduct

Once the trial design has been confirmed and the trial has started, the recommended dose level for the next patient is determined as follows:

i) Obtain available data on the patients currently in the trial;
ii) Update the estimated DLT probabilities at each dose using the model;
iii) Write a brief report detailing the model’s dose recommendation, along with estimates of DLT probabilities at all doses and any other quantities of interest; and
iv) If necessary, hold a meeting of the dose setting committee (DSC), or safety review committee (SRC), to formally decide whether to use the model’s recommendation or recommend a different dose (based on additional non-DLT toxicity data). The DSC is made up of researchers, clinicians, and members of the trial management group. The committee members attend dose decision meetings in person or via teleconference, and advise how the trial should proceed based on the safety data accrued during the trial. Dose transition pathways can be computed for one or more future cohorts [51] to aid the DSC in their recommendations.

Interim trial results should be reported to assist the DSC in decision-making. The results of interest fall into two categories: observed trial data, such as the grades and types of adverse event experienced by each patient and the number of adverse events that are classed as DLTs; and probabilistic results inferred from the dose-toxicity model.

### Report contents

Observed trial data results can be presented in simple frequency tables. A table of all observed adverse events as rows, with toxicity grades as columns, should be populated by the number of patients that experienced each adverse event of a particular grade. For example, if using the National Cancer Institute’s Common Terminology Criteria for Adverse Events (NCI CTCAE) grading system [66], low grades (e.g., 1 and 2) can be combined, as may higher grades (3 and 4) if, say, any grade 3 or higher adverse event is classed as a DLT. Any observed fatalities, classified as grade 5 adverse events, must be reported separately. Some trial publications divide these data across dose levels, providing a more accurate breakdown of which doses adverse events were observed at. For probabilistic results, we recommend providing the estimated (mean/median) probability of DLT per dose level with some measurement of variation or confidence/credible interval, either in a table or graph.

### Software for updating models and producing reports

Several software packages have been developed for designing, conducting, and analysing dose-finding studies using rule-based designs and the CRM (Table 2). These include software packages for popular statistical programs (e.g., R and Stata), as well as stand-alone programs with point-and-click user interfaces, some of which are freely available online. Many of these packages include tools for generating skeletons and dose labels under different dose-toxicity models and for simulating and conducting trials using likelihood-based and Bayesian methods. Help files are available for all programs, and most are provided with examples.

**Table 2:** Software for designing, simulating, and conducting dose-finding trials using rule-based designs and the CRM

Name	Host/Institution	Software/Stand-alone	Free/Commercial	Rule-based/Model-based	Description
bcrm [88]	CRAN	R	Free	Both	Design, run, and simulate trials using the CRM and 3 + 3 design
dfcrm [18]	CRAN	R	Free	Model-based	Design, run, and simulate trials using the CRM and Time-to-event CRM
crmPack [89]	CRAN	R	Free	Both	Design, run, and simulate trials using the CRM (includes other model-based designs, joint toxicity-efficacy modelling)
crm [90]	IDEAS (RePEc)	Stata	Free	Model-based	Run a single trial using the CRM
MoDEsT [91]	Lancaster University	Stand-alone (online)	Free	Model-based	Design, run, and simulate trials using the CRM
Bayesian CRM for phase I trials [92]	University of Virginia	Stand-alone (online)	Free	Model-based	Design, run, and simulate trials using the CRM
AplusB [93]	MRC Biostatistics Unit, University of Cambridge	Stand-alone (online)	Free	Rule-based	Compute exact operating characteristics for 3 + 3 and other rule-based designs
Center for Quantitative Sciences Calculator [94]	Vanderbilt University	Stand-alone (online)	Free	Both	Simulate trials using the CRM (uses bcrm [88] and dfcrm [18]) and other designs (rule-based/model-based)
CRMSimulator [95]	MD Anderson Cancer Center, University of Texas	Stand-alone	Free	Model-based	Simulate trials using the CRM
FACTS [96]	Berry Consultants	Stand-alone	Commercial	Both	Design program for phase I trials using the CRM, plus fixed and adaptive designs for phase II trials
ADDPLAN [97]	ICON PLC	Stand-alone	Commercial	Both	Design, simulate, and analyse trials using the CRM (includes methods for dose-response modelling)

*Abbreviations*: *CRAN* Comprehensive R Archive Network, *CRM* Continual Reassessment Method, *FACTS* Fixed and Adaptive Clinical Trial Simulator, *MoDEsT* Model-based Dose-Escalation Trials, *RePEc* Research Papers in Economics

## Results

To provide a sensible starting design that may be calibrated following simulation studies and investigator discussions, we recommend choosing initial trial parameters from the following options:

- Dose levels: between 4 and 8 levels;
- TTL: between 5% and 50%, but appropriate for the expected adverse events listed in the DLT definition, disease type and patient population;
- Prior guess of MTD: this dose should have prior estimate of DLT risk close or equal to the TTL;
- Model: power or logistic; one parameter is sufficient, but two parameter models are also used;
- Skeleton: use appropriate data from previous studies and clinical experience to specify prior DLT risks all doses; if not possible for all, consider specifying for some key doses (e.g. prior MTD, lowest dose, highest dose) and interpolate for levels in between. If challenging to do this, given prior guess of MTD and model choice, use the skeleton calibration approach of Lee and Cheung [36];
- Inference: if a run-in stage is required before using the model, likelihood or Bayesian methods can be used; otherwise, a Bayesian approach in a one-stage design can be used with either informative or uninformative priors depending on the availability of suitable data;
- Cohort size: between 1 and 3 patients, but no more than maximum number of available patients divided by number of dose levels;
- Safety rules: no-dose skipping, start at dose no larger than prior MTD, possibly the lowest dose;
- Stopping rules: terminate the trial for safety if there is high chance (e.g. at least 90%) that the risk of DLT at the lowest dose level is greater than the TTL. Consider adding additional stopping criteria if warranted by simulations and investigators.

Though recommendations from literature and experience are useful, case studies of published CRM trials are valuable learning tools. We present two real trials that used the CRM to identify the MTD of new cancer therapies; one trial using a one-stage Bayesian approach and another using a two-stage likelihood-based approach.

### Bayesian CRM: ssHHT in AML

Lévy et al. [67] conducted a dose-finding study to find the MTD of subcutaneous semi-synthetic homoharringtonine (ssHHT) given intravenously in patients with advanced acute myeloid leukaemia. Investigators planned to examine five dose levels of ssHHT (0.5, 1, 3, 5, and 6 mg/m^2^/day), and specified a TTL of 33%, or 0.33. The investigators chose a Bayesian CRM approach for the trial [68]. They used a one-parameter logistic model and placed an exponential prior distribution with a mean of 1 (and therefore variance of 1) on the slope parameter and fixed the intercept to be 3 (see Table 1). The prior for the slope parameter and fixed intercept were chosen after extensive simulation studies to ensure the model was suitable [personal correspondence with study statistician]. They based their skeleton (0.05, 0.10, 0.15, 0.33, and 0.50) on data from China, where a non-synthetic form of the molecule was used in practice. Dose labels were calculated using the skeleton and prior mean estimate of the model parameter.

During the trial, the posterior estimates for the probability of DLT at each dose were computed, and the next cohort received the dose with an estimated probability of a DLT closest to the TTL. Patients were dosed in three-person cohorts. The trial was to be terminated if adding another cohort of three patients would not change the estimate of the probability of a DLT at the MTD by more than 5%.

**Fig. 6:**
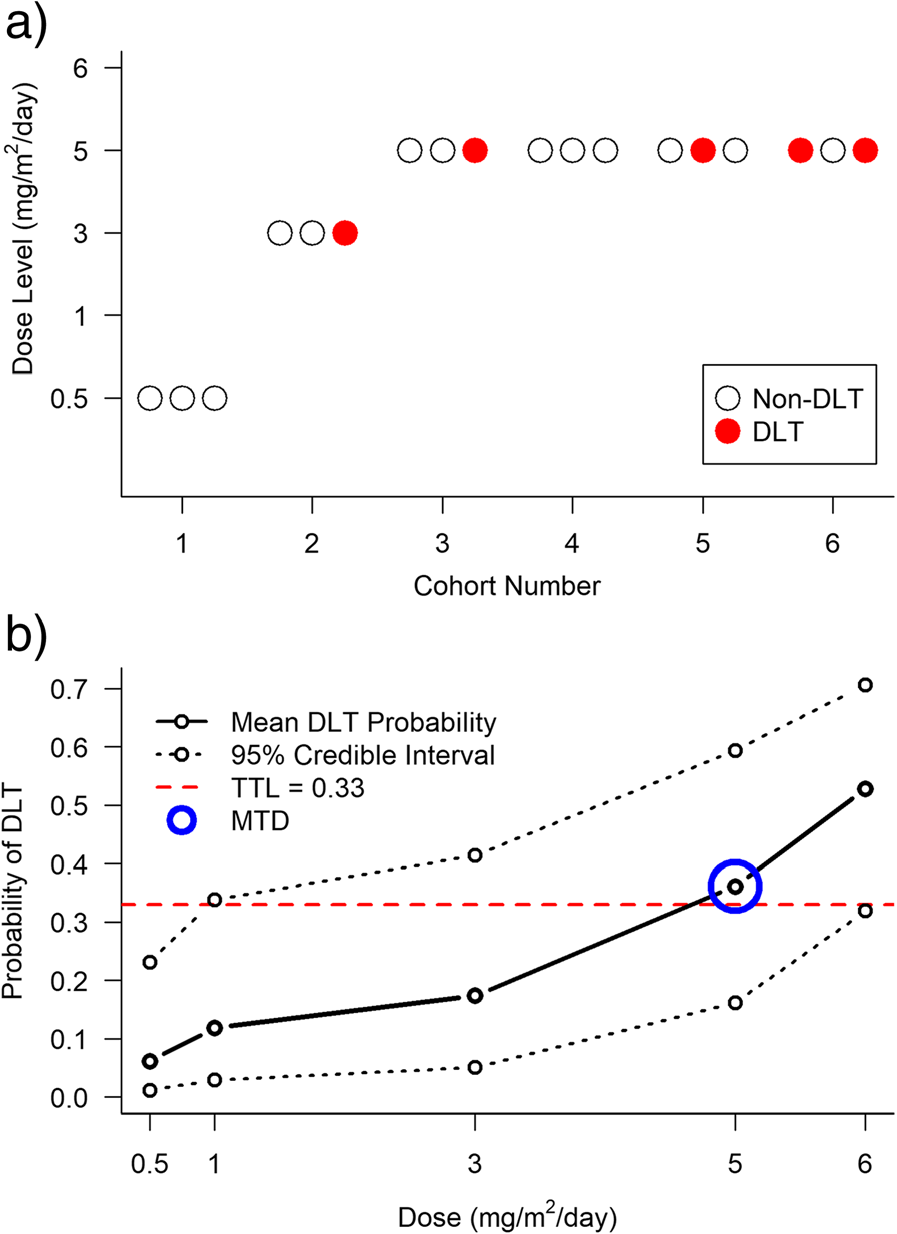
Results from the dose-finding trial of ssHHT in patients with advanced acute myeloid leukaemia [63] a) Trial conduct and DLTs observed. b) Final posterior mean estimates of DLT probabilities and 95% credible intervals (2.5th and 97.5th percentiles).

After observing no DLTs in the first cohort, who received 0.5 mg/m^2^/day, the model recommended the largest dose (6 mg/m^2^/day) for the next cohort. The investigators were not comfortable with this escalation and chose to dose the next cohort at 3 mg/m^2^/day. After one DLT out of three patients at 3 mg/m^2^/day, the next three patients were recommended to receive 5 mg/m^2^/day. The trial was terminated after treating 18 patients, as per the pre-specified stopping rule. Twelve patients received 5 mg/m^2^/day, four of whom experienced DLTs. At the end of the trial, the posterior estimates of DLT probabilities were 0.06, 0.12, 0.17, 0.36, and 0.53. As 5 mg/m^2^/day had a posterior estimate probability of a DLT closest to the TTL, it was selected to be the MTD (Fig. 6). Although we cannot say if fewer or more patients would have been recruited to the trial under a 3 + 3 design, the 3 + 3 design would have taken longer to reach the MTD level (nine patients dosed below the MTD, rather than six), and fewer patients would have been dosed at the MTD level during the trial (no more than six patients).

### Likelihood-based CRM: rViscumin in solid tumours

Paoletti et al. [69] conducted a trial to find the MTD of the lectin rViscumin given intravenously in patients with solid tumours. The dose levels to be investigated were 10, 20, 40, 100, 200, 400, 800, and 1600 ng/kg, with additional dose increases of 800 ng/kg. DLT was defined as any haematological grade 4 or non-haematological grade 3+ adverse event as per the NCI Common Toxicity Criteria Version 2 [70], with the exclusion of nausea, vomiting, or fever that could be rapidly controlled. The TTL was fixed at 20%, or 0.20.

The investigators implemented a two-stage likelihood-based CRM design, with a one-parameter power model for the dose-toxicity relationship. In the first stage, individual patients were dosed at increasing dose levels. The starting dose of 10 ng/kg was taken as 1% of the MTD in dogs. If a grade 2+ non-DLT adverse event was observed in one of these patients, another two patients were given that dose. If none of the three patients experienced a DLT, the first stage escalation continued. The model-based design stage was initiated when the first DLT was observed. Using a dose skeleton that was specified after the first DLT occurred (as it was not required during the first stage), dose labels were created for each dose. The estimates for the probability of a DLT at each dose were calculated using maximum likelihood methods and the next patient was given the dose with an estimated DLT probability closest to the TTL, subject to the constraint that no untested dose level could be skipped. Patients were dosed in single-patient cohorts, since low incidence of toxicity was expected, and the current patient was fully observed before the next patient was allocated to a dose. Although they did not state a planned sample size, the trial was to be terminated if the probability that the next five patients would be given the same dose level exceeded 90%.

**Fig. 7:**
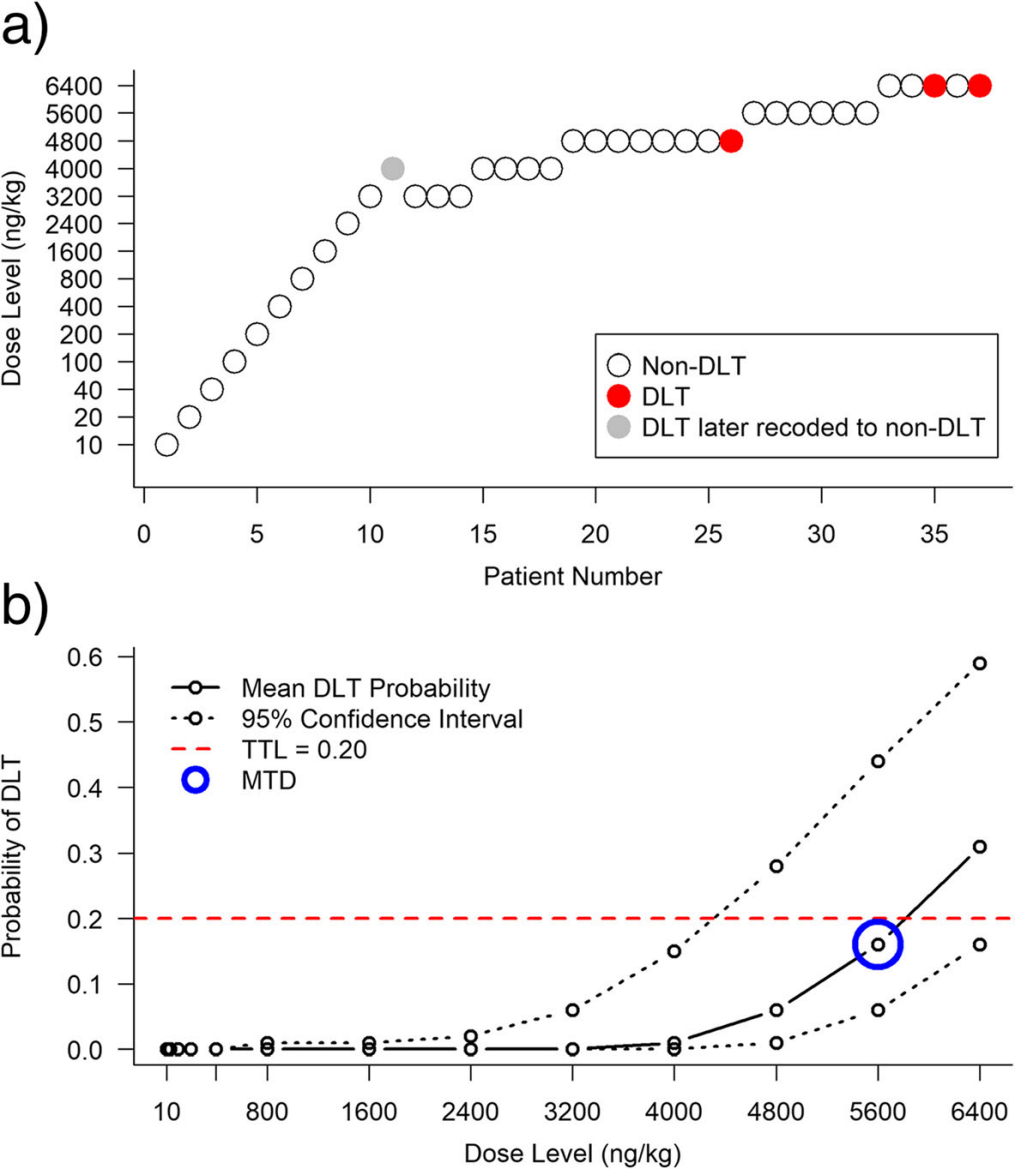
Results from the dose-finding trial of rViscumin in patients with solid tumours [65] **a**) Trial conduct and DLTs observed. **b**) Final mean estimates of DLT probabilities and 95% confidence intervals.

The first 10 patients, dosed at 10, 20, 40, 100, 200, 400, 800, 1600, 2400, and 3200 ng/kg respectively, had no moderate toxicity or DLTs. Patient 11, dosed at 4000 ng/kg, experienced a DLT (grade 3 asthenia), and from here the CRM design was used to make dose escalation/de-escalation recommendations, with oversight from the SRC. After estimating the model parameter, dose level 10 (3200 ng/kg), which had an estimated probability of DLT equal to 18%, was selected for patient 12. After patient 26 experienced a DLT (grade 3 transaminitis) at 4800 ng/kg, the SRC met to discuss dose allocation for patient 27. Upon review, the SRC recoded the DLT observed in patient 11 to a non-DLT, as it was resolved the same day it occurred. The SRC decided, given the revised estimates of DLT probability and the type of DLT observed, to dose patient 27 at the escalated dose of 5600 ng/kg (probability of DLT estimated as 21%). The trial was terminated after 37 patients were treated, 3 of whom had DLTs (patient 26 at 4800 ng/kg, patients 35 and 37 at 6400 ng/kg; all grade 3 transaminitis). The MTD was deemed to be 5600 ng/kg, with an estimated probability of DLT of 0.16 (95% confidence interval = (0.06, 0.44)). Figure 7 shows the conduct of the trial and the final estimates for the probability of a DLT with 95% confidence intervals. If a 3 + 3 design were used in this trial, at least 36 patients would have been dosed below the MTD. By using a two-stage CRM design, the sample size was reduced and the initial data from patients 1–10 were also used in dose-escalation decisions.

## Discussion

The CRM was first published in 1990. Its use in clinical trials, although increasing over time, remains low. Rogatko et al. [4] found 20 (1.6%) of 1235 phase I trials published between 1991 and 2006 used model-based designs, while a recent review found 92 (5.4%) of 1712 trials published between 2008 and 2014 used model-based designs, 59 (64.1%) of which used the CRM [5]. The infrequent use of the CRM is at odds with the mounting evidence that the CRM is better than the 3 + 3 design, both for estimating the MTD and for assigning more patients in the trial at the MTD. The example trials presented here show the Bayesian and likelihood-based CRM both dosed fewer patients at levels below the eventual MTDs than the 3 + 3 design, and dosed most of the patients recruited to the trial at or close to the MTD.

To encourage the uptake of the CRM in practice, we have provided a structured framework for designing, conducting and analysing phase I dose-finding trials using the CRM. We have separated the design stage into its core steps and, where possible, offered recommendations based on experience, the literature, simulation studies and published trials. There are several software packages and online applications available with supporting help files that can be used to design and simulate trials using the CRM, and we have also provided template text and tables that may be used in trial protocols and reports. However, the primary asset for designing a phase I trial with a model-based design is a trained statistician. Whilst more time and effort may be required during trial set-up than for a rule-based design, particularly for the first CRM study a trial team embarks on, these costs will decrease over time as experience increases. With respect to the authors’ host institutions, there are no standard operating procedures (SOPs) in place for designing CRM trials. Currently it is the expertise and judgement of the statistician(s), as well as the collaborative relationship between the study statistician(s) and clinical investigators, that are used to design the trial. The work by Yap et al. on designing the Viola trial (which used a CRM design) is a clear example of this in action [51]. However, with time, it may be the case that formal SOPs are introduced.

In this paper, we have only dealt with the simple case of a binary DLT endpoint that is fully observable in all patients. However, the CRM can be modified to deal with more nuanced endpoints and more complex trials, such as time-to-event outcomes [71–73], multiple toxicity grades [74, 75], joint toxicity and efficacy outcomes [76, 77], combinations of drugs [7], dose- and schedule-finding [78, 79], and patient covariates [80]. Like trials that use rule-based designs, dose-expansion cohorts can be added at the estimated MTD in a CRM-designed trial to obtain additional data on efficacy and tolerability [81–87].

## Additional file

Additional file 1: Appendices. (DOCX 260 kb)

## Abbreviations

CRAN: Comprehensive R Archive Network
CRM: Continual Reassessment Method
DLT: Dose-Limiting Toxicity
DSC: Dose Setting Committee
FACTS: Fixed and Adaptive Clinical Trial Simulator
MRC: Medical Research Council
MTD: Maximum Tolerated Dose
NCI CTCAE: National Cancer Institute’s Common Terminology Criteria for Adverse Events
RePEc: Research Papers in Economics
SRC: Safety Review Committee
ssHHT: semi-synthetic homoharringtonine
TTL: Target Toxicity Level
