# Supplementary material for: How to design a dose-finding study using the continual reassessment method
Source: BMC Med Res Methodol. 2019 Jan 18;19:18. doi: 10.1186/s12874-018-0638-z (PMC6339349; doi:10.1186/s12874-018-0638-z)
Supplement: Supplementary file 1 — Appendices. (DOCX 260 kb) [file 12874_2018_638_MOESM1_ESM.docx]

**Appendices**

**A Calculating the dose-toxicity skeleton**

In Table A1, we show calculations to compute dose labels from the prior skeleton shown in Figure 3, assuming a two-parameter logistic model.

**Table A1: Process of obtaining dose labels from a skeleton**

| **Dose level** | 1 | 2 | 3 | 4 | 5 | 6 |
| --- | --- | --- | --- | --- | --- | --- |
| **Dose (mg/m^2^)** | 10 | 20 | 40 | 80 | 120 | 160 |
| **Prior DLT probability (skeleton)** | 0.02 | 0.065 | 0.10 | 0.20 | 0.33 | 0.65 |
| **Calculation for dose label** | $\frac{ln(\frac{0.02}{1-0.02}) - 2}{exp(1)}$ | $\frac{ln(\frac{0.065}{1-0.065}) - 2}{exp(1)}$ | $\frac{ln(\frac{0.10}{1-0.10}) - 2}{exp(1)}$ | $\frac{ln(\frac{0.20}{1-0.20}) - 2}{exp(1)}$ | $\frac{ln(\frac{0.33}{1-0.33}) - 2}{exp(1)}$ | $\frac{ln(\frac{0.65}{1-0.65}) - 2}{exp(1)}$ |
| **Dose label** | -2.43 | -1.72 | -1.54 | -1.25 | -1.00 | -0.51 |

Two-parameter logistic model used with prior average parameter values β_1_ = 2 and β_2_ = 1.

**B Skeleton choice**

Skeleton probabilities can be automatically calculated using the dfcrm package [1] in R:

install.packages("dfcrm")

library(dfcrm)

TTL<-0.30 # Target Toxicity Level is 30%

delta<-0.05 # Want DLT risk at MTD to be between 25% and 35%

ndoses<-5 # Five dose levels

mtd<-4 # Prior belief is that dose level 4 is the MTD

model.choice<-"empiric" # We will use the power (empiric) model

skeleton<-getprior(target = TTL, halfwidth = delta, nlevel = ndoses, nu = mtd, model = model.choice)

skeleton

# Output given: [1] 0.06251978 0.12252936 0.20395601 0.30000000 0.40181944

We assume a Normal prior distribution for model parameter *β* with mean 0 (and so median 0) and standard deviation 1. We can plot the skeleton (prior median dose-toxicity relationship) and the 90% credible interval over the dose labels to see what our prior looks like and, if necessary, alter it to better reflect clinical prior belief (Figure B1).

prior.beta<-0 # Prior mean/median of model parameter ‘beta’

dose.labels<-skeleton^(1/exp(prior.beta)) # Get dose labels from skeleton (see Table 1).

beta<-rnorm(n = 1e6, mean = prior.beta, sd = 1) # Prior distribution ‘beta’

out.list<-lapply(1:ndoses, function(z) (dose.labels[z])^(exp(beta))) # List of prior distributions for probability of DLT per dose level

ci.limits<-c(0.05, 0.95)

cred.intervals<-sapply(1:ndoses, function(z) quantile(out.list[[z]], ci.limits)) # Prior 5% and 95% percentiles for probability of DLT

plot(dose.labels, skeleton, type = "b", las = 1, lwd = 2,
xlim = 0.05*c(round(range(dose.labels)/0.05)), ylim = c(0, 1), xlab = "Standardised Dose", ylab = "Probability of DLT", lty = 1) # Plot skeleton relationship (x-axis limits rounded to nearest 0.05)

lines(dose.labels, cred.intervals[1,], type = "b", lty = 3, lwd = 2)

lines(dose.labels, cred.intervals[2,], type = "b", lty = 3, lwd = 2)

abline(h = TTL, lty = 3, col = "red", lwd = 2)

abline(h = c(TTL-delta, TTL+delta), lty = 3, col = "red", lwd = 1)

legend(dose.labels[1], 1, lty = c(1, 3, 2, 3), pch = c(21, 21, NA, NA), pt.bg = c("white", "white", NA, NA), lwd = rep(2, 4), col = c("black", "black", "red", "red"), legend = c("Skeleton", "90% Credible Interval", "TTL", "TTL+/-0.05"))

***Figure B1: Prior distribution for the probability of DLTs across dose labels***


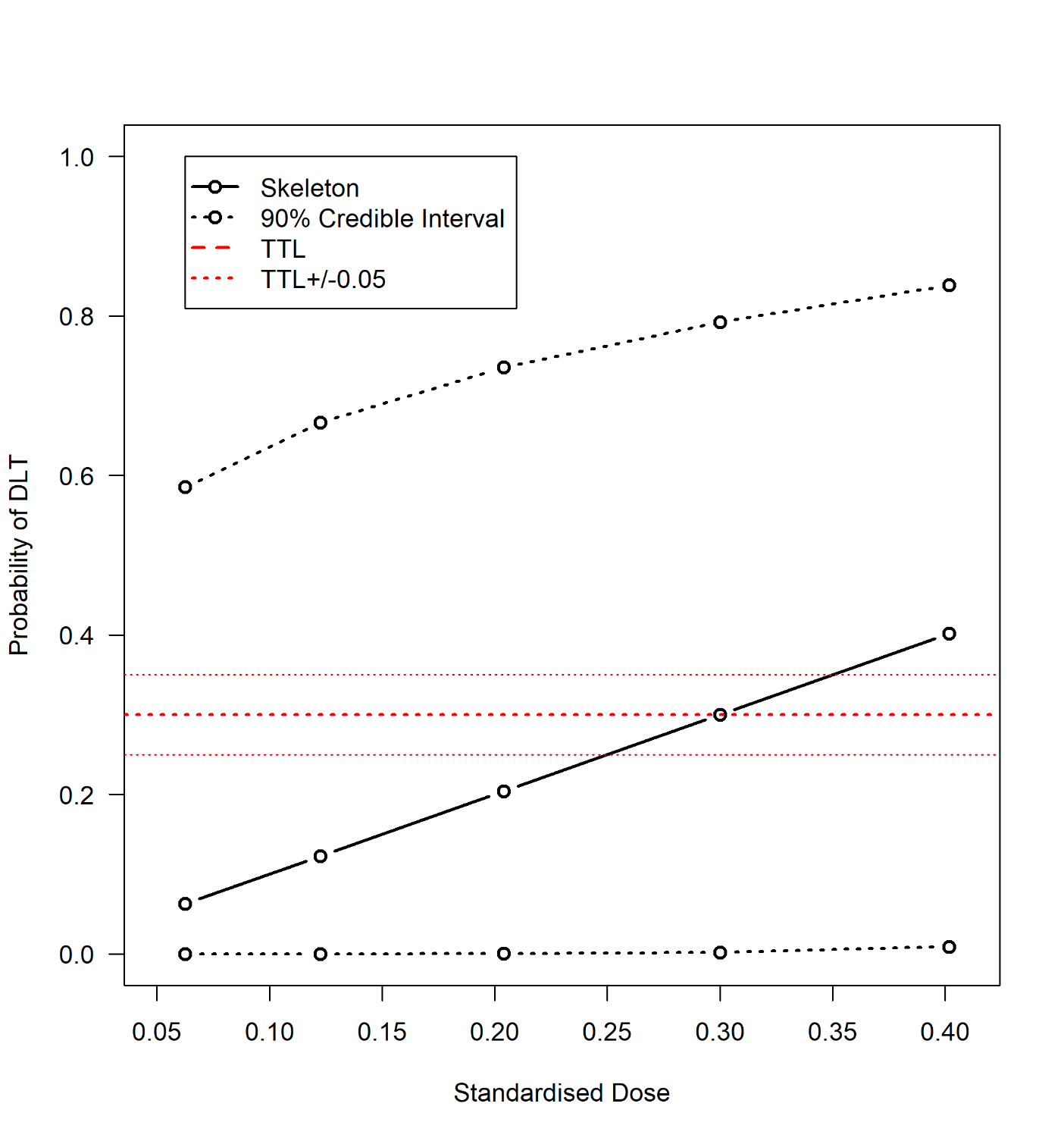


*Computed using the “getprior” function of the dfcrm package*[1]*.*

**C Template description of the CRM for a trial protocol**

The following template statement can be used to describe the CRM, with design parameters for a specific trial inserted in the relevant sections. These statements are to be used as guidelines for specifying the design of a dose-finding trial and should be verified by a statistician with experience in using model-based designs in dose-finding trials.

*“This dose-finding study will be conducted using the continual reassessment method (O’Quigley et al, 1990), a model-based design that informs how the dosage of <treatment name> should be adapted for the next patient cohort based on past trial data. We use a <one-parameter/two-parameter> <power/logistic> model to describe the relationship between the dose of <treatment name> and the probability of observing a DLT. The MTD is the largest dose that has an estimated probability of a DLT <less than/closest to> a target toxicity level of <TTL>. Patients will be dosed in cohorts of <cohort size>, with a maximum available sample size of <maximum sample size>. The trial will terminate when <insert details of stopping rules for trial here>.”*

*“Reference: O'Quigley J, Pepe M, Fisher L. Continual reassessment method: a practical design for phase 1 clinical trials in cancer. Biometrics. 1990;46(1):33-48.”*

For a one-stage Bayesian CRM, the following description can be added:

*“For each dose level of <treatment name>, prior average risks of DLTs have been specified (the skeleton) with the help of the clinical team. The prior distribution on the model parameter(s) is <insert details of prior distribution(s) here>. Table C1 shows the skeleton, dose labels, and <mean/median> and 90% credible interval for the prior risk of a DLT at each dose. After each patient cohort, the prior risks of DLTs will be updated with all available trial data by the trial statistician and the posterior risks of DLTs will inform the <dose setting committee/equivalent term> as to which dose is to be recommended for the next cohort. <List any dose-skipping/escalation constraints here>.”*

For a two-stage CRM, the following description can be added:

*“The design is formed of two stages. For each dose level of <treatment name>, prior average risks of DLTs will be specified (the skeleton) with the help of the clinical team (see Table <C1 if Bayesian, C2 if likelihood-based>). In the first stage, cohorts of <cohort size for first stage> patients will receive increasing dose levels of <treatment name> until the first DLT is observed. If the first <insert number of patients> patient(s) experience a DLT, the trial will be terminated early for safety. Once we observe at least one non-DLT response and at least one DLT response, the second stage of the design commences and the model parameters are estimated by the trial statistician using <likelihood-based/Bayesian> methods. After this, the estimated risks of DLTs at each dose will inform the <dose setting committee/equivalent term> as to which dose is to be recommended for the next cohort. <List any dose-skipping/escalation constraints here>.”*

Example formats for Table C1 and Table C2 (mentioned in descriptions above) are given below.

**Table C1: Prior <mean/median> risk of a DLT and 90% credible interval for each dose level**

| **Dose** | Dose 1 | … | Dose *k* |
| --- | --- | --- | --- |
| **Skeleton** | *p*_1_ | … | *p_k_* |
| **Dose labels** | *d*_1_ | … | *d_k_* |
| **Prior <mean/median>** | mean_1_/median_1_ | … | mean*_k_*/median*_k_* |
| **90% credible Interval** | 5^th^ and 95^th^ percentiles | … | 5^th^ and 95^th^ percentiles |

**Table C2: Skeleton and dose labels for likelihood-based CRM design**

| **Dose** | Dose 1 | … | Dose *k* |
| --- | --- | --- | --- |
| **Skeleton** | *p*_1_ | … | *p_k_* |
| **Dose labels** | *d*_1_ | … | *d_k_* |

**References**

1. Cheung YK. Dose Finding by the Continual Reassessment Method. Chapman & Hall/CRC Biostatistics Series, Taylor and Francis; 2011.
